# The neutrophil-to-lymphocyte ratio is associated with adverse outcomes in patients with anti-neutrophil cytoplasmic antibody-associated vasculitis

**DOI:** 10.3389/fimmu.2026.1780204

**Published:** 2026-03-26

**Authors:** Yunan Li, Zirui Liu, Di Zhang, Ziyi Sun, Haomiao Zhang, Liyuan Xie, Hongshan Chen, Chenxi Cai, Yuanyuan Li, Junya Jia, Pengcheng Xu

**Affiliations:** Department of Nephrology, Kidney Disease Medical Center, General Hospital, Tianjin Medical University, National Key Clinical Specialty, Tianjin Key Medical Discipline, Tianjin, China

**Keywords:** all-cause death, ANCA-associated vasculitis, end-stage renal disease (ESRD), neutrophil-to-lymphocyte ratio (NLR), prognosis

## Abstract

**Background:**

The neutrophil-to-lymphocyte ratio (NLR) is a convenient biomarker reflecting systemic inflammation and immune balance. While its prognostic value is established in other diseases, its role in predicting long-term outcomes in patients with ANCA-associated vasculitis (AAV) remains unclear. This study aimed to investigate the association between NLR and organ involvement, all-cause mortality, and end-stage renal disease (ESRD) in AAV patients.

**Methods:**

We conducted a retrospective study of 532 patients first diagnosed with AAV at Tianjin Medical University General Hospital between June 2012 and June 2024. The primary outcome was all-cause mortality, and the secondary outcome was ESRD. Logistic regression was used to assess associations between NLR and organ involvement. Restricted cubic spline (RCS), Cox proportional hazards regression, Kaplan-Meier survival analysis, time-dependent receiver operating characteristic (ROC) and subgroup analysis were employed to analyze the association between NLR and outcomes in AAV patients.

**Results:**

At baseline, patients with higher NLR level exhibited more severe inflammation, worse renal function, and higher disease activity (all *P* < 0.05). After adjustment for age and sex, NLR was independently associated with the presence of fever (adjusted OR 1.03, 95% CI 1.00–1.06, *P* = 0.042) and renal dysfunction (adjusted OR 1.04, 95% CI 1.01–1.07, *P* = 0.021) at diagnosis. RCS analysis revealed a nonlinear relationship between NLR and all-cause mortality, with a threshold of 10. After multivariable adjustment, patients in the high NLR group (NLR > 10) had a 77% increased risk of mortality compared to those in the low NLR group (adjusted HR 1.77, 95% CI 1.17–2.68, *P* = 0.007). NLR was not significantly associated with the risk of ESRD (adjusted HR 1.26, 95% CI 0.73–2.18, *P* = 0.400). The difference in the proportion of infection-related mortality between the high and low NLR groups was not statistically significant (60.0% vs 50.4%, *P* > 0.05).

**Conclusion:**

A nonlinear relationship with a saturation effect was observed between NLR and all-cause mortality in AAV patients. An elevated NLR served as an independent risk factor for adverse outcomes. This simple biomarker could be valuable for risk stratification in AAV patients.

## 1 Introduction

Antineutrophil cytoplasmic antibody (ANCA)-associated vasculitis (AAV) is a systemic disorder characterized by necrotizing vasculitis of small vessels, which can affect multiple organ systems, including the kidneys, upper and lower respiratory tracts, skin, eyes, sinuses, and peripheral nerves (1, 2). Previous studies report that AAV patients have a 5-year survival rate of 70%-78%, with delayed or inadequate treatment leading to a significant mortality burden (3, 4). Furthermore, persistent inflammatory responses play a pivotal role in the pathogenesis and progression of AAV (5). Consequently, the identification of accessible and robust biomarkers for the early stratification of high-risk patients is crucial for reducing AAV-related mortality and delaying the deterioration of renal function.

The neutrophil-to-lymphocyte ratio (NLR) is a simple and cost-effective laboratory marker that reflects both systemic inflammation and immune status in patients, representing the balance between lymphocyte-mediated adaptive immune responses and neutrophil-mediated innate immune responses. NLR has demonstrated prognostic potential in various conditions, including malignancies (6), cardiovascular diseases (7), and autoimmune disorders (8).

In recent years, there has been increasing interest in the clinical utility of NLR in AAV. Liu and colleagues observed that elevated NLR was not only positively correlated with the Birmingham Vasculitis Activity Score (BVAS) but also served as an independent predictor of concurrent infection in patients with newly diagnosed AAV (9). Similarly, a retrospective study by Fonseca and colleagues confirmed that high NLR at diagnosis independently predicted severe infection during the early phase of immunosuppressive therapy (10). The predictive value of NLR regarding long-term prognosis remains controversial. A study focusing on Chinese patients with myeloperoxidase (MPO)-AAV identified high NLR as an independent risk factor for all-cause mortality (11). However, Ahn and colleagues reported that while high NLR was associated with disease severity and relapse risk, no significant association with mortality risk was observed (12). These discordant findings may be attributable to relatively small sample sizes or heterogeneity in patient subtypes in previous studies. Moreover, whether a non-linear relationship exists between NLR and adverse outcomes in AAV requires further clarification.

Accordingly, our study, based on a large cohort of 532 patients with newly diagnosed AAV at Tianjin Medical University General Hospital, aims to investigate the associations between NLR and organ involvement, as well as all-cause mortality and ESRD, with the aim of providing more precise evidence-based medical evidence to facilitate clinical prognostic assessment for patients with AAV.

## 2 Materials and methods

### 2.1 Study population and design

This retrospective study enrolled 532 patients who were first diagnosed with AAV at Tianjin Medical University General Hospital and hospitalized between June 2012 and June 2024. Patient identification was strictly based on the 2012 Revised International Chapel Hill Consensus Conference Nomenclature for ANCA-associated vasculitis (13). The exclusion criteria were as follows: (1) presence of active infection; (2) the coexistence of hematological diseases; (3) diagnosis of malignant tumor or other autoimmune diseases; and (4) missing essential clinical or laboratory data. The research workflow was illustrated in **Supplementary Figure S1**. This study was conducted in accordance with the Declaration of Helsinki and was approved by the Ethics Committee of Tianjin Medical University General Hospital (Approval No.: IRB2025-KY-270). The requirement for written informed consent was waived due to the retrospective nature of the study.

### 2.2 Clinical and laboratory data

At the time of initial diagnosis, baseline demographic, clinical manifestations and laboratory data were collected from AAV patients, including age, gender, white blood cell (WBC), neutrophil, lymphocyte, and platelet counts; erythrocyte sedimentation rate (ESR), C-reactive protein (CRP), albumin (ALB), blood urea nitrogen (BUN), D-dimer, serum creatinine (Scr), estimated glomerular filtration rate (eGFR) (14), complement 3 (C3), complement 4 (C4), 24-hour urinary protein quantification, and other relevant indicators. All data were obtained prior to any therapeutic intervention.

Enzyme-linked immunosorbent assay was performed to detect ANCAs, including MPO and proteinase 3 (PR3). Renal dysfunction was defined as an eGFR of < 60 ml/min/1.73 m^2^ (15). Disease activity was assessed using the 2003 BVAS (16). The equation to calculate the NLR was neutrophil count/lymphocyte count.

### 2.3 Study outcome

The primary outcome was all-cause mortality and the secondary outcome was ESRD. For in-hospital deaths, comprehensive clinical records were reviewed. For out-of-hospital deaths, information was obtained through follow-up telephone interviews with family members. Causes of death were categorized into two groups: infection-related and non-infection-related. ESRD was defined by dialysis dependence for more than three months or receipt of a kidney transplant. Two independent physicians, blinded to the NLR data, adjudicated the death and ESRD based on this compiled information. Any discrepancies were resolved by consensus.

### 2.4 Statistical analysis

Continuous variables with normal distributions were presented as mean ± standard deviation (SD) and compared using Student’s t-tests. Skewed continuous variables were expressed as median (interquartile range, IQR) and analyzed via non-parametric tests. Categorical data were reported as percentages and chi-square or Fisher’s exact tests were utilized for group comparisons. Multiple imputation by chained equations (MICE) (17) was performed for variables with < 20% missing data. Missingness patterns are shown in **Supplementary Figure S2**.

Baseline characteristics were compared between groups stratified by the median NLR value (4.09) for descriptive purposes. The association between the NLR and organ involvement was assessed using logistic regression models, with results presented as odds ratios (ORs) with 95% confidence intervals (CIs). The multivariable model was adjusted for age and sex. The linear or non-linear relationships between NLR and the risks of all-cause mortality and ESRD in AAV patients were evaluated using restricted cubic splines (RCS) with three knots in Cox regression models, adjusted for age, sex, BVAS, eGFR, CRP, and ANCA subtype (2). Patients were stratified into low NLR and high NLR groups using a threshold of 10, which was derived from RCS. Kaplan-Meier survival analysis compared clinical outcome incidence between groups. Univariable and multivariable Cox proportional hazards models assessed associations between NLR and outcomes, with results reported as hazard ratios (HRs) and 95% CIs. The multivariable Cox models were adjusted for age, sex, BVAS, eGFR, CRP, and ANCA subtype. To compare the predictive accuracy for all-cause mortality of the NLR against its individual components (neutrophil count and lymphocyte count) and other clinical predictors, we constructed time-dependent receiver operating characteristic (ROC) curves and calculated the corresponding area under the curve (AUC) at 3 months, 0.5, 1, and 3 years of follow-up.

All analyses were performed using R software (version 4.5.1), with statistical significance defined as *P* < 0.05.

## 3 Results

### 3.1 Baseline patient characteristics

Of the 532 patients with AAV, stratified by a median NLR of 4.09, baseline characteristics are shown in **Table 1**. The cohort had a median age of 66.00 years (IQR: 60.00, 71.00), and 55.1% were female. Patients with an NLR > 4.09 were significantly older and had a lower proportion of females compared to those with an NLR ≤ 4.09. The group with NLR > 4.09 exhibited a more pronounced inflammatory state, with significantly elevated leukocyte counts, CRP, ESR, and D-dimer, together with lower hemoglobin and albumin levels (all *P* < 0.001). This group also presented with significantly worse renal function (higher Scr, lower eGFR, and greater proteinuria) and higher disease activity (BVAS) at baseline (all *P* < 0.05). No significant differences were found in comorbidities, ANCA subtypes, or complement levels.

**Table 1:** Baseline characteristics of 532 AAV patients.

Characteristics	All patients (n=532)	NLR ≤ 4.09 (n=266)	NLR > 4.09 (n=266)	*P* value
Age (years) (median, IQR)	66.00 (60.00, 71.00)	64.00 (57.25, 70.00)	67.00 (62.00, 72.00)	<0.001
Sex (female), n (%)	293 (55.1)	164 (61.7)	129 (48.5)	0.003
Smoking history, n (%)	205 (38.5)	96 (36.1)	109 (41.0)	0.285
Drinking history, n (%)	110 (20.7)	49 (18.4)	62 (23.3)	0.200
Diabetes, n (%)	87 (16.4)	44 (16.5)	44 (16.5)	1.000
Hypertension, n (%)	217 (40.8)	104 (39.1)	113 (42.5)	0.480
Cardiovascular Disease, n (%)	79 (14.8)	32 (12.0)	47 (17.7)	0.088
WBC (× 10^9^/L) (median, IQR)	8.94 (6.64, 11.66)	7.33 (5.90, 9.38)	10.63 (8.38, 13.74)	<0.001
Neutrophil (× 10^9^/L) (median, IQR)	6.34 (4.55, 9.01)	4.72 (3.65, 6.04)	8.63 (6.40, 11.18)	<0.001
Lymphocyte (× 10^9^/L) (median, IQR)	1.48 (1.03, 1.94)	1.80 (1.47, 2.29)	1.09 (0.83, 1.49)	<0.001
Monocyte (× 10^9^/L) (median, IQR)	0.59 (0.43, 0.76)	0.54 (0.41, 0.69)	0.65 (0.47, 0.83)	<0.001
Platelet (× 10^9^/L) (median, IQR)	273.50 (208.75, 356.25)	253.00 (200.50, 329.25)	294.00 (222.25, 377.75)	<0.001
Hb (g/L) (mean, SD)	103.69 (23.96)	110.35 (24.93)	97.03 (20.96)	<0.001
ESR (mm/h) (median, IQR)	50.00 (35.00, 60.00)	45.00 (31.00, 58.00)	55.00 (43.25, 62.00)	<0.001
Serum ANCA subtyping, n (%)				0.752
Double negative	13 (2.4)	9 (3.4)	4 (1.5)	
MPO-ANCA	423 (79.5)	197 (74.1)	225 (84.6)	
PR3-ANCA	85 (16.0)	51 (19.2)	35 (13.2)	
Double positive	11 (2.1)	9 (3.4)	2 (0.8)	
C3 (mg/dl) (median, IQR)	92.70 (77.30, 110.00)	91.20 (78.38, 107.00)	93.40 (76.93, 111.75)	0.626
C4 (mg/dl) (median, IQR)	23.00 (18.38, 28.50)	22.70 (18.70, 28.48)	23.00 (18.10, 28.37)	0.567
CRP (mg/dl) (median, IQR))	2.76 (0.64, 8.44)	0.95 (0.34, 3.61)	7.74 (2.10, 11.43)	<0.001
D-Dimer (ng/ml) (median, IQR)	1586.50 (696.25, 3216.50)	901.00 (473.25, 1854.25)	2604.50 (1390.00, 4570.00)	<0.001
Scr (μmol/L) (median, IQR)	96.50 (63.00, 238.50)	86.00 (58.00, 170.25)	114.50 (68.25, 325.75)	<0.001
eGFR (mL/min/1.73m^2^) (median, IQR)	67.07 (20.82, 113.56)	79.62 (31.94, 123.00)	54.48 (14.46, 100.56)	<0.001
ALB (g/L) (median, IQR)	32.00 (27.75, 36.00)	35.00 (30.25, 38.00)	30.00 (26.00, 33.00)	<0.001
Proteinuria (mg/24h) (median, IQR)	520.00 (257.78, 1254.00)	436.00 (221.00, 1255.50)	553.50 (320.00, 1219.50)	0.027
BVAS (median, IQR)	15.00 (11.00, 19.00)	14.00 (10.00, 18.00)	15.00 (12.00, 20.00)	<0.001

NLR, neutrophil-to-lymphocyte ratio; IQR, interquartile range; WBC, white blood cell counts; Hb, hemoglobin; SD, standard deviation; ESR, erythrocyte sedimentation rate; ANCA, antineutrophil cytoplasmic antibody; MPO, myeloperoxidase; PR3, proteinase 3; C3, complement 3; C4, complement 4; CRP, C reactive protein; Scr, serum creatinine; eGFR, estimated glomerular filtration rate; BUN, blood urea nitrogen; ALB, albumin; BVAS, Birmingham Vasculitis Activity Score.

### 3.2 Association between NLR and organ involvement at diagnosis in AAV patients

We employed logistic regression analysis to assess the association between NLR and organ involvement at initial diagnosis (**Table 2**). In univariable analysis, a higher NLR was significantly associated with the presence of fever (OR 1.03, 95% CI 1.01–1.06, *P* = 0.028), renal dysfunction (OR 1.04, 95% CI 1.01–1.08, *P* = 0.011), and lung involvement (OR 1.13, 95% CI 1.04–1.25, *P* = 0.010).

**Table 2:** Logistic regression analysis of organ involvement according to NLR.

Organ involvement	Number (percent)	Crude OR (95% CI)	*P* value	Adjusted OR (95% CI)	*P* value
Fever	215(40.4)	1.03 (1.01-1.06)	0.028	1.03 (1.00-1.06)	0.042
Renal dysfunction	247(46.4)	1.04 (1.01-1.08)	0.011	1.04 (1.01-1.07)	0.021
Lung	473 (88.9)	1.13 (1.04-1.25)	0.010	1.07 (0.99-1.18)	0.173
Circulatory system	20 (3.8)	1.01 (0.98-1.04)	0.256	1.01 (0.97-1.04)	0.308
Nervous system	61 (11.5)	1.00 (0.96-1.02)	0.983	1.00 (0.96-1.02)	0.884
Digestive system	29 (5.5)	1.01 (0.97-1.03)	0.493	1.01 (0.97-1.03)	0.510
Cutaneous	36 (6.8)	0.98 (0.91-1.02)	0.593	1.00 (0.93-1.03)	0.859
Eyes/Mucosa	40 (7.5)	0.97 (0.89-1.01)	0.372	0.98 (0.90-1.02)	0.531
ENT	67 (12.6)	1.01 (0.98-1.03)	0.482	1.01 (0.98-1.03)	0.409
Muscle/joint	116 (21.8)	0.99 (0.96-1.01)	0.475	0.99 (0.96-1.02)	0.632

The multivariate logistic regression model was adjusted for age, sex.

OR, odds ratio; CI, confidence interval; ENT, ear, nose, and throat.

After adjustment for age and sex, the associations with fever (adjusted OR 1.03, 95% CI 1.00–1.06, *P* = 0.042) and renal dysfunction (adjusted OR 1.04, 95% CI 1.01–1.07, *P* = 0.021) remained statistically significant. The association with lung involvement was attenuated and no longer significant in the adjusted model (adjusted OR 1.07, 95% CI 0.99–1.18, *P* = 0.173). We found no significant associations between NLR and involvement of the circulatory, nervous, digestive, or other systems.

### 3.3 Association between NLR and long-term outcomes in AAV patients

**Figure 1:**
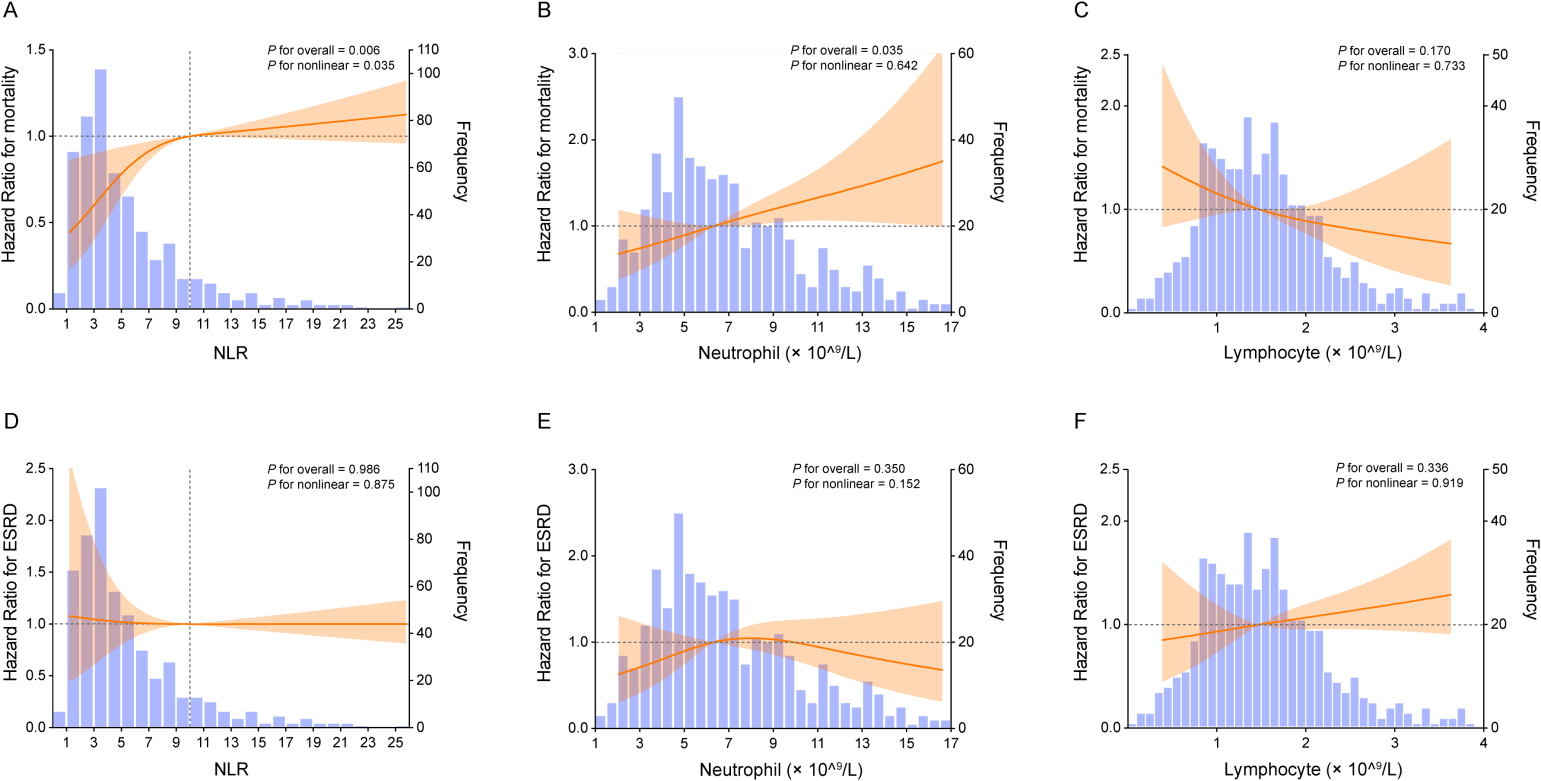
Restricted cubic spline (RCS) illustrating the associations of NLR with all-cause mortality **(A)** and ESRD **(D)**; neutrophil count with all-cause mortality **(B)** and ESRD **(E)**; and lymphocyte count with all-cause mortality **(C)** and ESRD **(F)**. HRs were adjusted for age, sex, BVAS, eGFR, CRP, and ANCA subtype.

The 532 enrolled patients with AAV were followed up for a median of 31.08 months. During this period, 156 deaths from any cause and 90 ESRD events were recorded. After adjusting for key clinical variables, RCS analysis revealed a nonlinear relationship between NLR and all-cause mortality (*P* for overall = 0.006, *P* for nonlinear = 0.035; **Figure 1A**) in AAV patients. In contrast, no significant association was observed between NLR and the risk of progressing to ESRD (*P* for overall = 0.986, *P* for nonlinear = 0.875; **Figure 1D**). A positive correlation was observed between NLR levels and mortality to the left of the inflection point (NLR = 10), indicating that the risks of all-cause mortality significantly increased with rising NLR. However, beyond the point, the association between NLR and the risks of mortality attenuated, indicating a potential saturation effect at elevated NLR levels. Meanwhile, an association was observed between neutrophil counts and all-cause mortality (P for overall = 0.035; **Figure 1B**). However, no significant association was found between lymphocyte counts and all-cause mortality. Furthermore, neither neutrophil counts nor lymphocyte counts were significantly associated with ESRD (P for overall > 0.05; **Figures 1B, C, E and F**).

**Figure 2:**
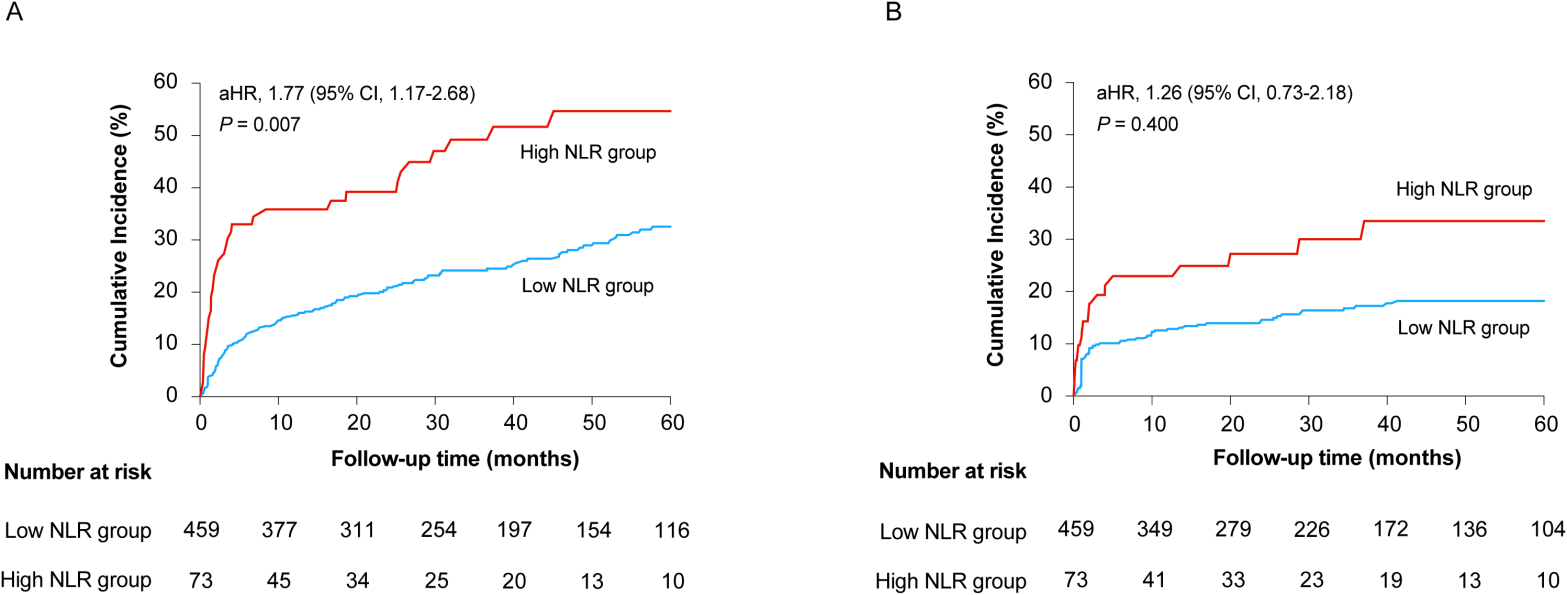
Kaplan-Meier curves comparing the cumulative incidence of all-cause mortality **(A)** and ESRD **(B)** between patients with high (>10) and low (≤10) NLR levels.

Based on the threshold identified by the RCS analysis, patients were stratified into low NLR (≤10) and high NLR (>10) groups. Kaplan-Meier survival analysis revealed that patients in the high NLR group had significantly higher rates of all-cause mortality (**Figure 2A**) and ESRD (**Figure 2B**) compared to those in the low NLR group. As shown in **Table 3**, after multivariable adjustment, patients in the high NLR group had a 77% increased risk of mortality compared to those in the low NLR group (adjusted HR 1.77, 95% CI 1.17–2.68, *P* = 0.007). For ESRD, while a significant association was observed in the univariable model (HR 2.05, 95% CI 1.24–3.41, *P* = 0.005), it was attenuated and became non-significant after adjusting for confounders (adjusted HR 1.26, 95% CI 0.73–2.18, *P* = 0.400).

**Table 3:** Risks of all-cause mortality and ESRD according to NLR levels in AAV patients.

Outcome	Low NLR group (n = 459)	High NLR group (n = 73)	Crude HR (95% CI)	*P* value	Adjusted HR (95% CI)	*P* value
All-cause mortality	121 (26.4)	35 (47.9)	2.38 (1.64, 3.48)	< 0.001	1.77 (1.17, 2.68)	0.007
ESRD	71 (15.5)	19 (26.0)	2.05 (1.24, 3.41)	0.005	1.26 (0.73, 2.18)	0.400

Adjusted for age, sex, BVAS, eGFR, CRP, ANCA subtype.

NLR, Neutrophil-to-lymphocyte ratio; ESRD, end stage renal disease; HR, hazard ratio; CI, confidence interval.

### 3.4 Comparative prognostic value of NLR over its components and other clinical predictors

**Figure 3:**
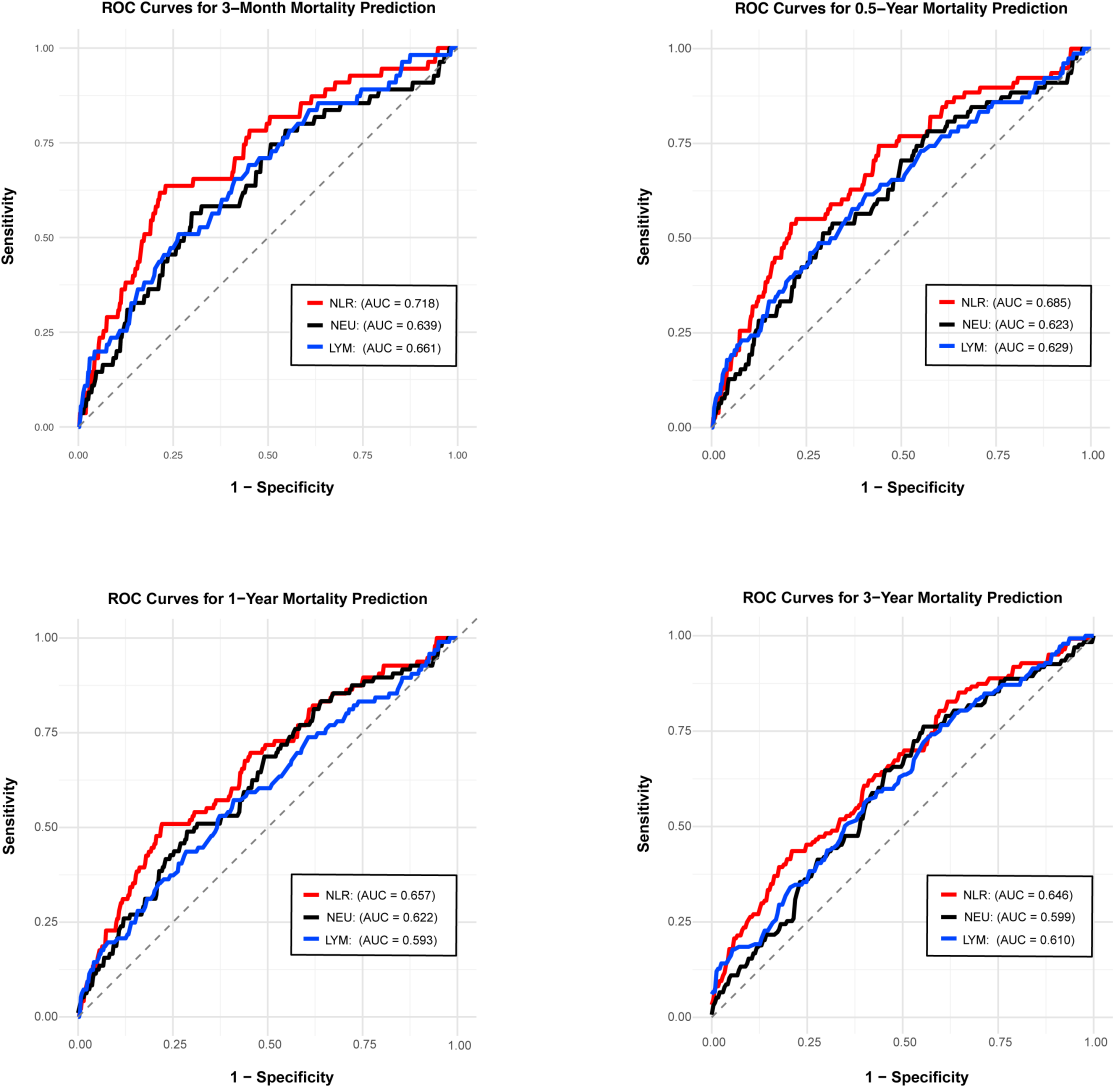
ROC analysis showing the prognostic performance of the NLR, absolute neutrophil count, and absolute lymphocyte count in predicting all-cause mortality at 3 months, 0.5, 1, and 3 years. NLR, neutrophil-to-lymphocyte ratio; NEU, neutrophil; LYM, lymphocyte.

Time-dependent ROC analyses at 3 months, 0.5, 1, and 3 years were performed to compare the prognostic performance of NLR, neutrophil count, and lymphocyte count (**Figure 3**). The AUC for NLR was higher than that for either neutrophil or lymphocyte alone at all time points (at 3 months: NLR AUC [0.718], neutrophil AUC [0.639], lymphocyte AUC [0.661]; at 0.5 years: NLR AUC [0.685], neutrophil AUC [0.623], lymphocyte AUC [0.629]; at 1 year: NLR AUC [0.657], neutrophil AUC [0.622], lymphocyte AUC [0.593]; at 3 years: NLR AUC [0.646], neutrophil AUC [0.599], lymphocyte AUC [0.610]). Furthermore, the AUC of NLR was consistently higher than those of BVAS and CRP across all evaluated time points (**Supplementary Table 1**).

### 3.5 Subgroup analyses

**Figure 4:**
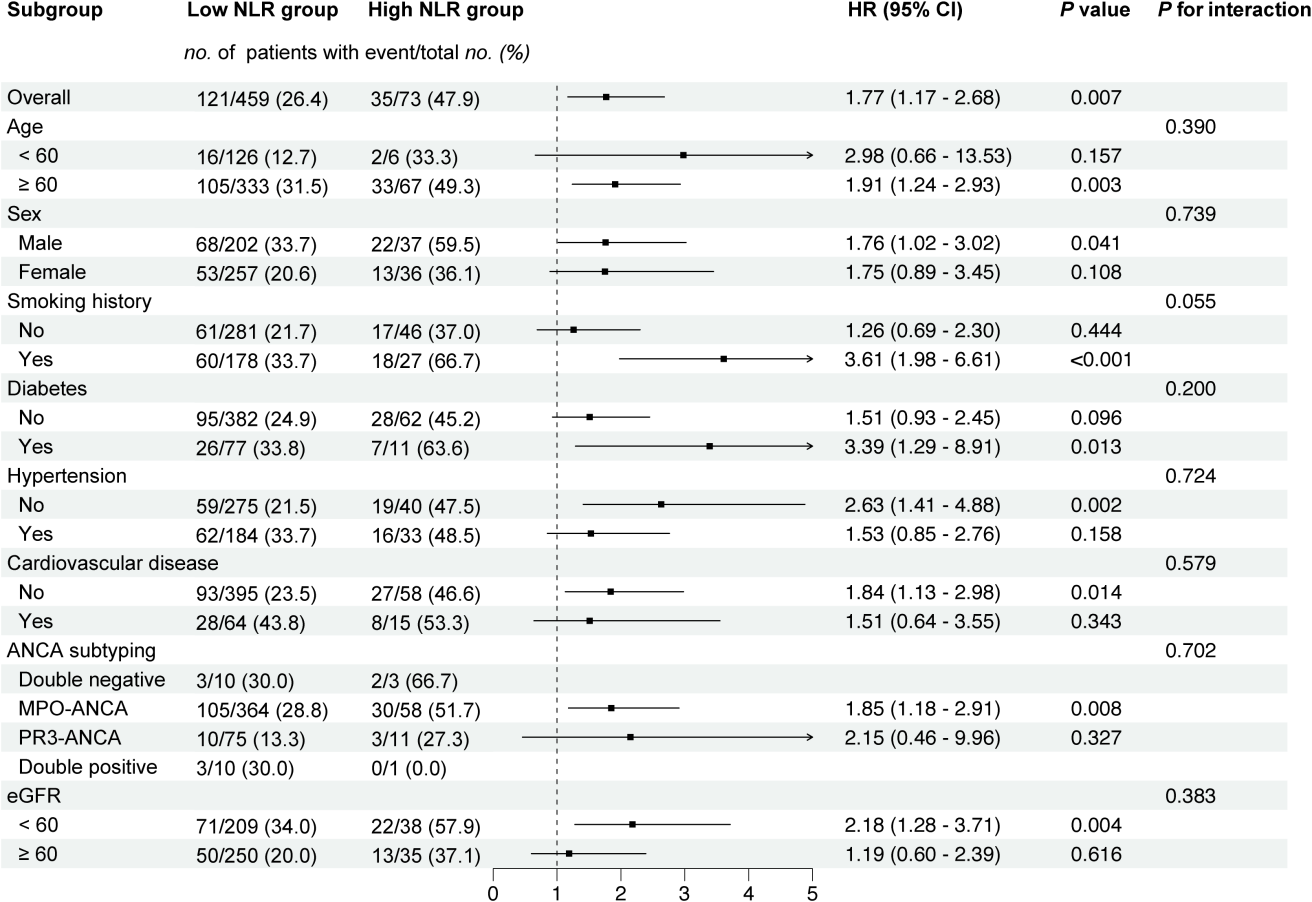
Forest plot of subgroup analysis of the association between NLR and all-cause mortality among AAV patients. HRs were adjusted for age, sex, BVAS, eGFR, CRP, and ANCA subtype. When analyzing age, adjustments were made for the other variables, and so on for subsequent analyses.

We conducted stratified analyses based on key clinical variables, including age, sex, smoking history, diabetes, hypertension, cardiovascular disease, ANCA subtype, and eGFR. As shown in **Figure 4**, the effects of NLR on the rate of death from any cause were consistent across the prespecified subgroups. There were no significant interactions between NLR and subgroup with respect to the death from any cause (all *P* for interaction > 0.05).

### 3.6 Association between NLR and causes of mortality in AAV patients

**Figure 5:**
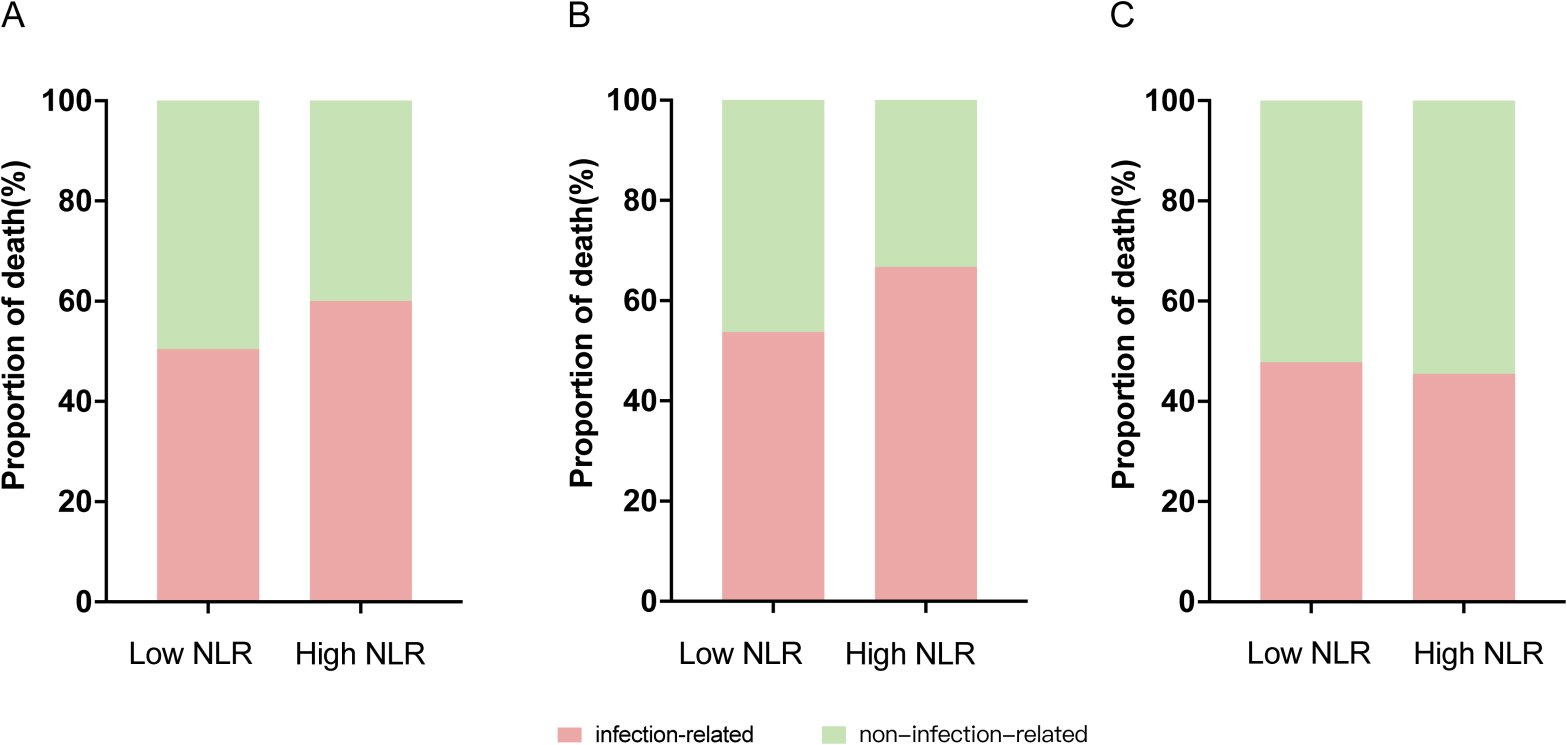
Distribution of primary causes of death stratified by baseline NLR levels among all deceased patients **(A)**, patients who died within 6 months **(B)**, and patients who died after 6 months **(C)**.

Among the 156 deceased patients (**Figure 5A**), no statistically significant difference in the distribution of causes of death was observed between the the high NLR group (n = 35) and the low NLR group (n = 121, *P* = 0.317). However, a clinically relevant trend was noted, with the high NLR group exhibiting a higher proportion of infection-related deaths (60.0% vs 50.4%).

This pattern was similarly observed among patients who died within 6 months (66.7% vs 53.7%, *P* = 0.285; **Figure 5B**). For late deaths (> 6 months), those in the high NLR group had a higher proportion of non-infection-related deaths compared with infection-related deaths (54.5% vs 45.5%; **Figure 5C**).

## 4 Discussion

In this large-scale retrospective cohort study of 532 newly diagnosed AAV patients, we evaluated the association of baseline NLR with organ involvement and long-term adverse outcomes, including all-cause mortality and ESRD. The results indicated that NLR is independently associated with fever and renal dysfunction. Notably, we identified a significant non-linear association between NLR and all-cause mortality, with a high NLR constituting an independent risk factor after multivariable adjustment. Furthermore, NLR demonstrated a modest improvement in predictive performance for all-cause mortality compared with neutrophil or lymphocyte counts alone.

As an inflammatory biomarker derived from two leukocyte subtypes, NLR has demonstrated prognostic value in various immune-mediated diseases, including rheumatoid arthritis (8), IgA nephropathy (18), and diabetic nephropathy (19). Conversely, Ahn et al. suggested limited relationship of NLR for mortality in AAV (12). Consistent with findings by Tian et al. (20), our study indicates that elevated NLR correlates with higher disease activity and increased mortality risk. However, leveraging a larger cohort, the present study is the first to elucidate the non-linear relationship between NLR and all-cause mortality in AAV. Previous studies largely presumed a linear relationship between the NLR and clinical outcomes (11), an assumption that may oversimplify the underlying pathophysiology. In contrast, our RCS analysis revealed a nonlinear dose-response relationship. Specifically, mortality risk exhibited a positive association with increasing NLR up to a threshold of 10, beyond which the risk plateaued. This observed “saturation effect” indicates that NLR values exceeding 10 are not associated with a further escalation in adverse outcome risk. This non-linear pattern provides a clinical insight: while rising NLR within the lower range signals increasing hazard, an extremely elevated NLR may represent a distinct pathophysiological state rather than a simple continuum of risk. However, we cannot rule out the possibility that apparent plateaus may arise from data sparsity at the distribution tails. The limited sample size in the high-NLR group may compromise the reliability of hazard ratio estimates in these regions.

Our data suggest that NLR predicts all-cause mortality better than neutrophil or lymphocyte counts alone. Neutrophils are the primary effector cells in AAV pathogenesis (21). The binding of ANCA to Fcγ receptors on neutrophils leads to neutrophil overactivation, resulting in excessive release of pro-inflammatory cytokines, reactive oxygen species (ROS), proteolytic enzymes, alongside the formation of neutrophil extracellular traps (NETs), directly causing endothelial cell injury (22–24). Conversely, lymphocytes generally reflect nutritional status and immune function. During the progression of inflammatory diseases, a relative reduction in lymphocyte counts suggests suppression or exhaustion of adaptive immunity, impairing the body’s ability to regulate inflammation (25). Furthermore, specific lymphocyte subsets, such as regulatory T cells, play a pivotal role in maintaining immune homeostasis and suppressing inflammation (26). In summary, as a combined index, NLR effectively reflects systemic inflammatory status and the balance between innate and adaptive immunity, thus providing valuable information for clinical assessment.

Evidence from multiple studies indicates that an elevated NLR is independently associated with the incidence and progression of chronic kidney disease (27–29), suggesting that the systemic inflammatory state it represents may be implicated in the initiation and aggravation of renal impairment. Notably, however, while high NLR was an independent predictor of mortality in our study, its association with ESRD lost statistical significance after adjusting for clinical variables, including age, renal function (eGFR) and disease activity (BVAS) (2). Nevertheless, the potential influence of NLR on renal endpoints warrants further consideration given the following constraints. First, the median follow-up duration of 31.08 months in our cohort may be insufficient to adequately capture the long-term renal outcomes in AAV patients. Second, the progression to ESRD depends directly on the severity of baseline renal histopathology and the response to subsequent immunosuppressive therapy, which were not fully captured in our models. Therefore, future prospective studies with extended follow-up periods and comprehensive inclusion of these established clinicopathological predictors are warranted to further clarify the relationship between NLR and long-term renal prognosis.

Previous investigations have demonstrated that infection is the predominant cause of mortality within the first year of diagnosis (47%), whereas active vasculitis itself accounts for a relatively smaller proportion of deaths (19%) (30, 31). Fonseca et al. identified high NLR as an independent risk factor for severe infection during the early phase of immunosuppressive therapy, thereby adversely affecting 1-year mortality (10). Although our analysis did not yield statistically significant differences in the distribution of causes of death across NLR groups, a numerical trend was observed, whereby infection-related mortality was higher in the high NLR group, particularly among patients with early mortality. This finding underscores the biological significance of NLR as a marker of systemic inflammation and immune dysregulation, suggesting the necessity for heightened clinical vigilance regarding infection in this patient population. The lack of statistical significance may be attributed to the limited number of fatal events; thus, larger cohort studies are warranted to further elucidate the association between NLR and specific causes of death.

This study has several limitations. First, as a single-center retrospective study, the results may be subject to selection bias, which could limit the generalizability of our findings. Second, our Cox regression models did not account for treatment-related variables (e.g., immunosuppressive regimens or glucocorticoid exposure), renal histopathology, or longitudinal proteinuria, which may limit the interpretability of the findings. Although we adjusted for potential confounding factors identified in previously published literature, residual confounding cannot be precluded. Finally, we only assessed the baseline NLR level and were unable to analyze the dynamic changes of NLR during treatment and its relationship with prognosis.

## 5 Conclusion

In summary, this study has established a non-linear association with a saturation effect between NLR and all-cause mortality. NLR was closely associated with baseline renal dysfunction and served as an independent risk factor for long-term adverse outcomes. This simple and widely available biomarker has the potential to become an effective tool for risk stratification and prognosis evaluation in AAV patients.

## Data Availability

The raw data supporting the conclusions of this article will be made available by the authors, without undue reservation.
