# Supplementary figures and images for: The neutrophil-to-lymphocyte ratio is associated with adverse outcomes in patients with anti-neutrophil cytoplasmic antibody-associated vasculitis
Source: Front Immunol. 2026 Mar 26;17:1780204. doi: 10.3389/fimmu.2026.1780204 (PMC13062306; doi:10.3389/fimmu.2026.1780204)

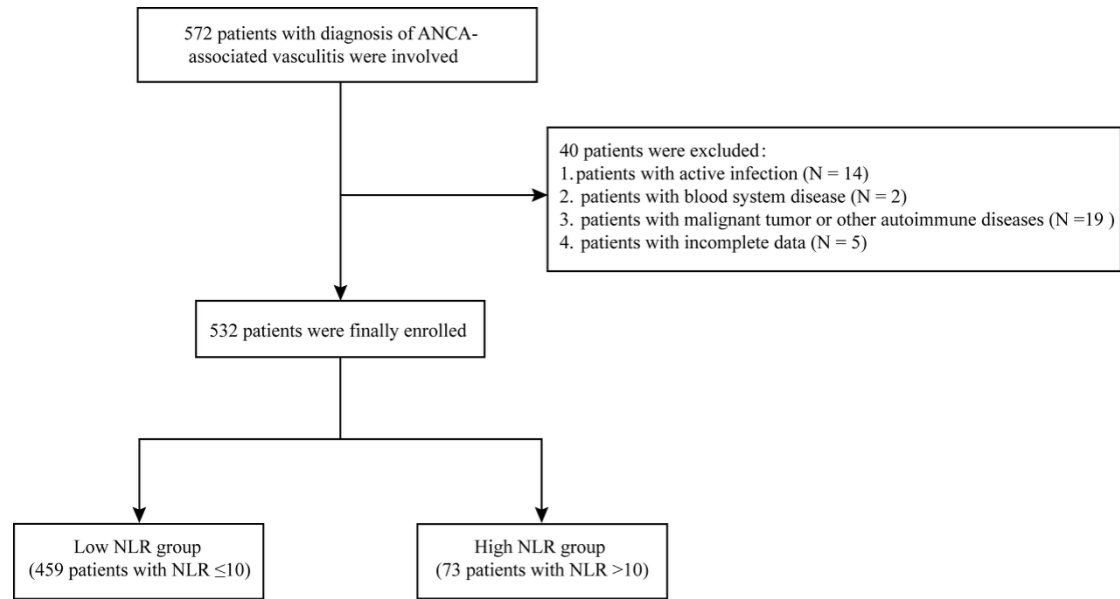

Figure S1. The flow chart of excluded patients.

Supplement: Supplementary file 1 [file DataSheet1.pdf]

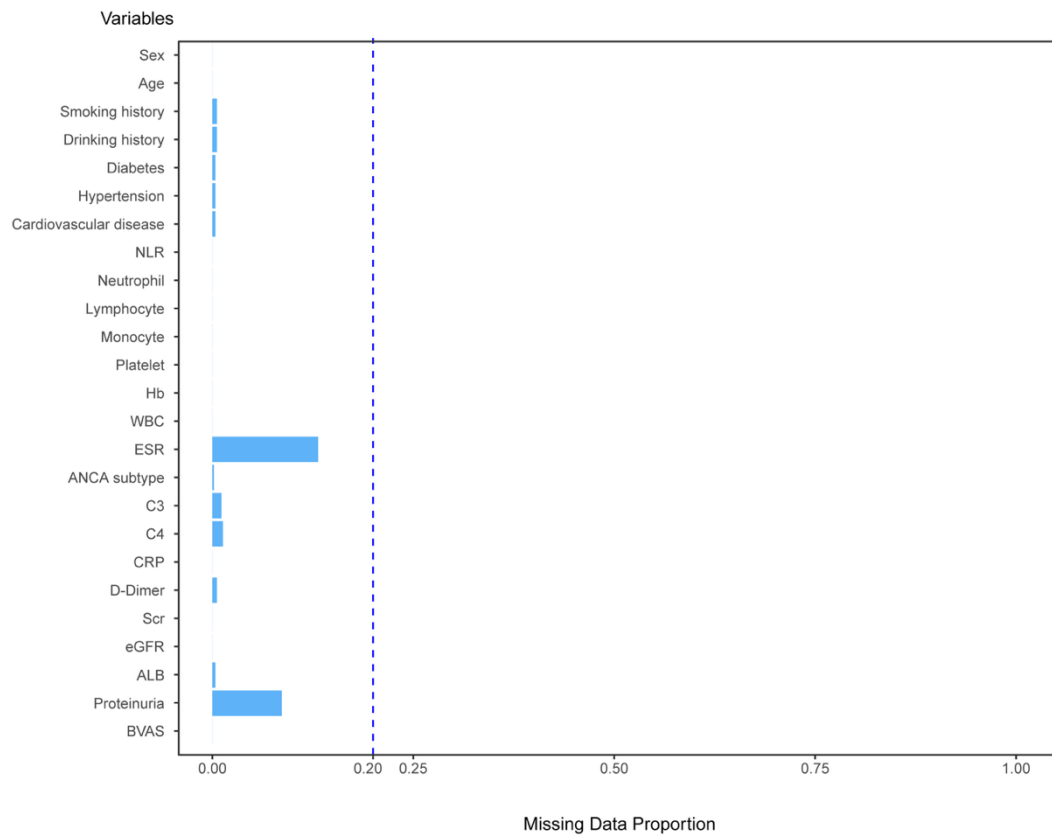

Figure S2. Missingness patterns in the dataset.

Supplement: Supplementary file 2 [file DataSheet2.pdf]
