# Supplementary material for: The neutrophil-to-lymphocyte ratio is associated with adverse outcomes in patients with anti-neutrophil cytoplasmic antibody-associated vasculitis
Source: Front Immunol. 2026 Mar 26;17:1780204. doi: 10.3389/fimmu.2026.1780204 (PMC13062306; doi:10.3389/fimmu.2026.1780204)
Supplement: Supplementary file 3 [file Table1.pdf]

Supplementary Table 1. ROC analysis showing the prognostic performance of the NLR and other predictors in predicting all-cause mortality at 3 months, 0.5, 1, and 3 years.

Parameters	3 months	0.5 years	1 year	3 years
NLR	0.718	0.685	0.657	0.646
Age	0.662	0.674	0.704	0.718
eGFR	0.725	0.710	0.678	0.647
BVAS	0.666	0.637	0.620	0.598
CPR	0.570	0.589	0.578	0.581

NLR, Neutrophil-to-lymphocyte; eGFR, estimated glomerular filtration rate; BVAS, Birmingham Vasculitis Activity Score; CRP, C reactive protein
